# Using Social Media for Peer-to-Peer Cancer Support: Interviews With Young Adults With Cancer

**DOI:** 10.2196/28234

**Published:** 2021-09-02

**Authors:** Allison J Lazard, Meredith K Reffner Collins, Ashley Hedrick, Tushar Varma, Brad Love, Carmina G Valle, Erik Brooks, Catherine Benedict

**Affiliations:** 1 Hussman School of Journalism and Media University of North Carolina at Chapel Hill Chapel Hill, NC United States; 2 Lineberger Comprehensive Cancer Center University of North Carolina at Chapel Hill Chapel Hill, NC United States; 3 School of Advertising and Public Relations The University of Texas at Austin Austin, TX United States; 4 Gryt Health New York, NY United States; 5 Department of Nutrition Gillings School of Global Public Health University of North Carolina at Chapel Hill Chapel Hill, NC United States; 6 Stanford University School of Medicine Stanford, CA United States

**Keywords:** cancer survivors, social support, peer groups, social media, young adults, pyscho-oncology, mobile phone

## Abstract

**Background:**

Web-based social support can address social isolation and unmet support needs among young adults with cancer (aged 18-39 years). Given that 94% of young adults own and use smartphones, social media can offer personalized, accessible social support among peers with cancer.

**Objective:**

This study aims to examine the specific benefits, downsides, and topics of social support via social media among young adults with cancer.

**Methods:**

We conducted semistructured interviews with young adults with cancer, aged between 18 and 39 years, who were receiving treatment or had completed treatment for cancer.

**Results:**

Most participants (N=45) used general audience platforms (eg, Facebook groups), and some cancer-specific social media (eg, Caring Bridge), to discuss relevant lived experiences for medical information (managing side effects and treatment uncertainty) and navigating life with cancer (parenting and financial issues). Participants valued socializing with other young adults with cancer, making connections outside their personal networks, and being able to validate their emotional and mental health experiences without time and physical constraints. However, using social media for peer support can be an emotional burden, especially when others post disheartening or harassing content, and can heighten privacy concerns, especially when navigating cancer-related stigma.

**Conclusions:**

Social media allows young adults to connect with peers to share and feel validated about their treatment and life concerns. However, barriers exist for receiving support from social media; these could be reduced through content moderation and developing more customizable, potentially cancer-specific social media apps and platforms to enhance one’s ability to find peers and manage groups.

## Introduction

Social media has the potential to provide critical social support for young adults with cancer [1,2]. Cancer has unique psychosocial impacts on young adults (aged 18-39 years), with unmet social support needs being a challenge for many [3-5]. Young adults with cancer struggle to find peers with their diagnoses and are often affected by social isolation, which is compounded by debilitating life disruptions such as extended absences from school and work [3,4,6,7]. Feelings of isolation may occur throughout their cancer experience and into survivorship [8,9]. Lack of social support among young adults with cancer is associated with poorer physiological and physical functioning, greater psychological distress, and reduced quality of life [10-12].

Many young adults with cancer report a need to meet peer survivors [3] and desires for peer connections via convenient, technology-based support options [13,14]. With 94% of young adults in the United States owning a smartphone [15], social media can leverage the power of networks and the ubiquity of smartphones for accessible, personalized social support [16]. Web-based social support can also uniquely contribute to well-being [17,18], especially among those unable or unwilling to receive face-to-face support [19]. Young adults may particularly benefit from peer support, defined as seeking or sharing emotional, informational, or instrumental support among other young adults with cancer via social media [20]. Young adults with cancer consistently express the need to connect with peers to feel less alone in their unique challenges, hear from others’ experiences for what to expect, and maintain or form their identity [3,14,21]—the need for support is potentially alleviated through synchronous or asynchronous social media connections among youth [17,22,23].

Social media also appears to increase social connectedness among young adults with cancer [24,25]. Peer-to-peer sharing on social media may meet unique, age-specific needs [26-29]; however, many still recognize the need to better understand how young adults with cancer use social media for social support [30,31]. Social media engagement can involve complex relational processes where young adults with cancer benefit most from reciprocal and responsive disclosures [32]; however, few have addressed upstream experiences that facilitate (or hinder) cancer-related conversations on social media. In addition, although many have analyzed the content (eg, social media posts) of cancer conversations [28,33,34], less attention has been given to why young adults with cancer decide to engage before messages are sent (or not sent). There is also a need to identify the downsides of peer support among individuals with cancer to generate possible solutions, as social media use can negatively influence well-being among young adults [35]. Previous studies have often focused on the positive impacts of social media use, with a limited focus on potential downsides in these novel approaches [29,36]. If it is known that emotions drive social connections on the web, including negative mental experiences (eg, isolation and fear) [30], then there is a need to provide more balanced approaches to understand why young adults are motivated to organically share their stories and drawbacks of peer support. Thus, for this study, we seek to examine specific benefits and downsides, along with motivations for specific topics of peer-to-peer social support when using social media from the lived experiences of young adults with cancer.

## Methods

### Participants and Procedure

We conducted semistructured interviews with young adults with cancer between April and May 2020. Participants were recruited via email and social media posts (Facebook, Instagram, and Twitter) from *Stupid Cancer*, one of the largest adolescent and young adult cancer advocacy organization in the United States. Emails and social media posts had a brief description of the study and incentives, eligibility recruitments (aged between 18 and 39 years and those either currently receiving treatment or have completed treatment for cancer), and how to contact the team. Potential participants emailed to confirm eligibility by stating their age and whether they had a cancer diagnosis and scheduled an interview. We received 163 initial responses and scheduled the first 45 eligible respondents for 30-minute interviews for “our study to learn more about what young adults affected by cancer look for in online support” (recruitment description). We planned to schedule additional interviews if we did not reach saturation, but these were not needed.

All interviews were conducted via Zoom by 2 researchers (AJL and MKRC) to allow for virtual participation. The day before the interview, participants received a web-based Qualtrics survey with written consent and demographic items, including age, gender, diagnosis, and treatment status. Before the interview, the researcher confirmed their consent and permission to record. This study was part of a larger data collection to better understand web-based social support. For this study, participants were first asked about social media they were currently using or had previously used for support, how these apps or platforms were helpful for making connections and support, what issues or topics they connected with others about, and any downsides of web-based social support, with questions adapted from our cancer support app research [29]. Then, all interviewees were asked about initiation or any changes to how they used web-based support and who they connected with, which is reported elsewhere [37]. Despite the vast disruptions to life for months to come from COVID-19, these interviews were planned and conducted before the scope was known, and the impact of the pandemic was not an explicit focus in the interviews. Each participant received a US $40 Amazon gift card. The University of North Carolina Institutional Review Board approved all procedures (#19-2715); all team members completed human-subjects training before the study.

### Data Analysis

#### Overview

Interviews were auto-transcribed using Zoom software and then cleaned for accuracy by 2 researchers listening to and manually correcting any errors in the transcripts (AH and TV). The same 2 researchers coded the transcripts with codes developed a priori for specific apps or platforms used and large *broad-brush* topics [38] about benefits, downsides or hesitations, and topics of support. Disagreements were reviewed, discussed by the research team, and then resolved independently by the first and last author (AJL and CB) [39,40]. All the members of the research team interpreted the individual codes into broader themes.

#### Statistical Methods

Descriptive statistics were conducted in SPSS 26 to analyze participant demographics.

## Results

### Overview

The participants (N=45) were mostly White (36/45, 80%) and female (33/45, 73%; Table 1). The average age was 31 (SD 5.56) years, and the average age at diagnosis was 26 (SD 6.95) years. One-third of the participants (15/45, 33%) reported breast cancer as their primary diagnosis. More than half of the participants (25/45, 56%) had completed treatment at the time of the interview. Participants resided in every geographic region of the United States.

The participants primarily reported the use of general audience platforms for cancer support. Almost all participants reported using Facebook, generally or through private groups, or Instagram at some point in their cancer timeline. Twitter use was organically mentioned by just over a tenth of young adults, and a few shared about using Snapchat, TikTok, or YouTube to seek out cancer stories. About a quarter of young adults referenced using cancer-specific social media (eg, Gryt Health and Caring Bridge) or web-based forums of national cancer advocacy organizations, such as Stupid Cancer or American Cancer Society. About 1 in 6 participants reported the use of video chat platforms, such as Zoom or Webex, which became popular in the COVID-19 pandemic. A few also highlighted texting cancer peers, sometimes via WhatsApp, and meeting through other means (eg, in-person meet-ups and support groups). Reported social media use was similar across age, gender, diagnoses, or treatment status; themes were also consistent unless noted below.

**Table 1:** Demographics of participants in this study (N=45).

Characteristics			Values
Current age (years), mean (SD)			31.00 (5.56)
Age at diagnosis (years), mean (SD)			26.39 (6.95)
**Gender, n (%)**			
	Male	12 (27)	
	Female	33 (73)	
**Race, n (%)**			
	Asian	3 (7)	
	Black or African American	6 (13)	
	White	36 (80)	
**Ethnicity, n (%)**			
	Hispanic or Latino or Latina	3 (7)	
	Non-Hispanic or Latino or Latina	42 (93)	
**Diagnosis^a^, n (%)**			
	Breast cancer	15 (33)	
	Hodgkin lymphoma	6 (13)	
	Leukemia	4 (9)	
	Sarcoma	3 (7)	
	Lung cancer	2 (4)	
	Brain tumor	2 (4)	
	Ovarian or uterine cancer	2 (4)	
	Colon or rectal cancer	2 (4)	
	Other cancer	9 (20)	
**Treatment status, n (%)**			
	In treatment	9 (20)	
	Ongoing therapies	10 (22)	
	Completed treatment	25 (56)	
**Region of residence in the United States, n (%)**			
	New England	1 (2)	
	Mid-Atlantic	12 (27)	
	South	9 (20)	
	Midwest	9 (20)	
	Southwest	5 (11)	
	West	9 (20)	

^a^According to the American Cancer Society [41], the leading cancer types in adolescents and young adults are thyroid cancer, breast cancer, lymphomas, leukemia, testicular germ cell tumors, melanoma, soft tissue and bone sarcomas, colon and rectum cancer, and uterine cervix cancer, followed by cancers at other sites.

### Topics of Web-Based Social Support

#### Overview

On social media, young adults commonly had conversations about medical information and navigating life with cancer when seeking or sharing support among peers. The participants noted the benefits and downsides of these conversations. Participants’ responses were edited for grammar and clarity.

#### Medical Information

Young adults with cancer often turn to peers on social media for medical information because they feel uncertain about future chemotherapy, treatments, and operations; what side effects to expect; how to manage side effects; and what comes after their current treatment. Many are often unsure whether their side effects and symptoms are normal or warrant provider care. Social media offers accessible ways to share and compare experiences, for example:

I think it’s a nice way for people to sort of manage their anxiety around the uncertainty of what’s to come. Because there’s a lot of it when you’re diagnosed.

Female, 32 years, breast cancer, ongoing therapies

Reading others’ cancer posts helps alleviate the fear of the unknown.

Participants also received and shared personal *tips and tricks* for dealing with symptoms and side effects, such as using over-the-counter or alternative medicine to help with nausea, swelling, and pain. Connecting with others to receive diagnosis-specific information was especially important. Some online groups are organized by diagnosis or treatment to allow young adults to seek relevant or cancer-specific peer support from informal mentors:

You kind of adopt someone to sort of, like, mentor you, if that makes any sense. And it’s not formal, it’s just when you feel like, oh, I really kind of like that person. I feel like they’re easy to talk to.

Female, 38 years, breast cancer, ongoing therapies

Other organizations facilitate mentorships between members with similar diagnoses that can lead to peer support:

I did start out with Imerman Angels, and since my mentor was on the West Coast and I’m at the East Coast, it was, you know, online. She introduced me to Twitter [cancer community].

Female, 38 years, brain tumor, completed treatment

Generally, participants found hashtags or search bars within private groups helpful for seeking out diagnosis-specific information.

Sometimes, advice-sharing with peers on the web extends to clinical information about drugs and treatments. Several participants used online groups or communities to *crowdsource* information, asking others about information from providers or web-based resources. One participant explained the following:

What I like from Facebook these days, especially, I use it as a crowdsourcing platform. So, when I have a question...I’ll have 12,000 people potentially. 12,000 answers.

Female, 38 years, brain tumor, completed treatment

Participants value obtaining information from peers because they are usually jargon-free and often feel more complete than provider information. For example, one participant said the following:

Your oncologist you know, will tell you all the side effects, but it’s very comforting and nice to hear actually come out of someone’s mouth that’s been on the medication...they tell you the raw deal of it, what it really is, or what they took for it, how long they’ve been on it, how long it’s worked for them. So, I find it very informative. It’s like a whole other side that like my oncologist doesn’t reveal to me.

Female, 33 years, breast cancer, ongoing therapy

Participants also turned to social media or online groups to express dissatisfaction with their doctors and seek recommendations for other providers:

Another thing we talked a lot with everybody about is doctor recommendations. That’s flying around a lot. Who you’re happy with, who you’re not happy with, who would you suggest people to stay away from.

Female, 38 years, breast cancer, ongoing therapies

Similarly, a breast cancer survivor shared how peer support influenced her choice of provider:

I didn’t really like the way they wanted to do my reconstruction. It was what they wanted, not what I wanted...that’s when the social media came into place where I could see what others had done and then you know what, what their outcomes look like from the doctors or the facilities. And so, I think it really helped me choose where I finally did go for my reconstruction....So, it wasn’t like, that, you know, it wasn’t like you were getting a doctored photo. It was like you’re getting a real-life situation.

Female, 38 years, breast cancer, completed treatment

#### Navigating Life With Cancer

Participants sought social support for navigating the impact of cancer on life, including their professional lives and personal relationships. Peer support on social media was often mentioned as helpful for managing work and career goals. One young adult turned to social media when having *continued issues* and needing advice on how others have handled returning to work (male, 32 years, colon cancer, in treatment). Another shared the following:

I found that I follow some women who are vocal about their cancer process and cancer diagnosis on Twitter and that’s been helpful because...I feel like [they are] in similar sort of professional situations as me...at a similar point in their career, which is important for me as someone who, you know, hopes to who knows eventually like enter [profession].

Female, 32 years, breast cancer, ongoing therapies

A small number of female participants used social media for peer support about dating and sex, including finding helpful mechanisms for coping:

One thing that I didn’t realize...there’s like a lot of funny cancer jokes...they really have helped me understand a lot of stuff and think, like, okay, I’m not the only one...they talk a lot about...different relationship stuff and dating.

Female, 29 years, breast cancer, ongoing therapies

The few parent participants shared about connecting for specific advice to talk to their children or support with parenting more broadly:

How do you explain it to them in a way that’s, you know, it’s not scary, but you’re also not lying to them, you know?

Female, 32 years, lung cancer, in treatment

One of the groups I’m in is a stage four moms group [with young kids]....And sometimes...that page is a little more not centered on cancer so much. But then there’ll be, “I hope I get to see them graduate,” and stuff like that.

Female, 29 years, breast cancer, ongoing therapies

Participants also received advice about managing the impact of cancer on their bodies. A few female participants who had completed treatment shared about supporting those with hair loss:

A *lot of the times it’s like, you don’t have to shave your head right in the beginning, you can wait or when it feels right to you...we all give each other like supportive feedback on it.* [Female, 24 years, Hodgkin lymphoma, completed treatment]

Peers also shared fitness or exercise support, advice about eating—albeit not always evidence-based nutrition—and support for fertility or other debilitating effects of treatment:

A lot of people have said that yoga seems to help them because it’s not a strenuous but it’s you know, working their muscles.

Female, 32 years, lung cancer, in treatment

There’s conflicting information out there...some say the keto diet...a plant-based diet...vegan style is the best...And then there’s a lot of people selling supplements, which can be really expensive and maybe a bit dangerous to take without any guidance from a medical professional.

Male, 29 years, sarcoma, completed treatment

They have a lot of like different groups for different specific things. I found the general colon cancer group to be sort of overwhelming because there was a lot of people in a lot of different situations, but when you [are] specifically looking for help with, like, neuropathy or fertility issues or people in the local area, it was nice to be able to filter it down to those things.

Male, 32 years, colon cancer, in treatment

Young adults, primarily those in posttreatment, also reported frequently discussing mental health among peers as a group or for individual support:

*They do like a mental health check post and everyone, they’ll say like, “how are you doing today” and we just allcomment*. [Female, 30 years, breast cancer, completed treatment]

I spoke to someone on Twitter because they had posted something about like, “is this normal, blah, blah?” And I, like, privately messaged them...“yes.” I was like, “I was at my most anxious, my most depressed, like, a year after treatment.”

Female, 29 years, Hodgkin lymphoma, completed treatment

Notably, in the initial months of a global pandemic, a few mentioned seeking advice for managing exposure to coronavirus while immunocompromised recommendations.

Both during and posttreatment, young adults turned to peers on social media for advice about the unique support needs introduced by cancer. Young adults sought out peers for navigating insurance or financial support:

They post things, they take polls on all different topics relating to cancer...like, you know, insurance “did your insurance cover this”

Female, 31 years, ovarian cancer, completed treatment

The big one for me is financial assistance...as far as the cancer that I have, I kind of know what it is, I kind of know if there’s a cure or not...the big thing for me with the cancer was financial assistance. What help was out there for people like me, going through this.

Male, 31 years, recurrent neuroblastoma, in treatment

Young adults also seek and share about cancer-related stigma:

Whenever I get on the group, [pain medicine shaming]’s like the hugest topic. And, you know, and we’re, you know, it’s a thing that makes us really angry too because we can’t help that we need pain medicine, you know?

Female, 36 years, breast cancer

Young adults rely on web-based social support to navigate their survivorship. Upon finishing treatments, young adults sought guidance by transitioning back to *normal* life. Describing feelings posttreatment, one participant said the following:

Oh my gosh, I am, like, broken, and traumatized. How am I supposed to go back [to life before cancer]? So that’s when I just needed, I guess, just to hear from others, like “Holy crap, this is really hard.”

Female, 28 years, Hodgkin lymphoma, completed treatment

Additional struggles include lasting impacts of *chemo brain*, facing survivor’s guilt, and coping with the deaths of friends with cancer.

Some young adults also enjoyed sharing non–cancer-related topics with their peers on the web. One participant explained the following:

It would be overwhelming if we constantly talked about cancer, cancer, cancer.

Female, 30 years, breast cancer, completed treatment

Many of these participants talked about shared hobbies and local bars and restaurants, providing recommendations for people nearby or temporarily around for treatment. These conversations are especially enjoyable among age-similar peers and can foster lasting friendships. However, not all participants perceived conversations as steering away from cancer; one participant said the following:

We started talking about other stuff, you know, just our daily life...But it seems it all still revolves around cancer-based things just because, I guess it’s such a big part of our life.

Female, 29 years, breast cancer, ongoing therapies

### Benefits of Web-Based Social Support

Beyond receiving information from peers for desired topics, participants noted several other benefits of web-based social support, including connecting with others, mental health and emotional benefits, and web-specific benefits.

#### Connecting With Others

Web-based social support can reduce loneliness via alternate ways of socializing. With cancer, meeting in person can be difficult because of being immunocompromised to exhaustion or pain and wanting to keep diagnoses private. Sometimes, friends and family members are afraid or nervous to talk about cancer. Many of these challenges become nonissues in online cancer-focused groups, where young adults can comfortably *lean on* peers in similar situations any time of day. This was especially helpful when needing to talk while having pain, when receiving difficult news from the doctor, or when hearing about a friend’s declining health. Many posttreatment participants use social media to *give back* and share support for those undergoing treatment—who join diagnosis-specific Facebook groups to find an informal mentor with a similar diagnosis. A few participants expressed how using web-based social support to combat loneliness became even more important during the COVID-19 pandemic, as many young adults with cancer were immunocompromised and needed to practice strict social isolation for their safety:

I mean, especially because I’m not leaving my house right now, so I like the Zoom being able to see people and being able to interact.

Female, 37 years, lung cancer, completed treatment

Some participants also mentioned how the pandemic created new opportunities for connecting with peers:

And I do like that during this time of COVID, my cancer center has been moving towards online activities that don’t have anything necessarily to do with cancer...I wish that had been more [popular before] because, sometimes you’re at home, recovering from a surgery and you’d like to be talking to other cancer patients, but you don’t have the stamina or mental energy to hear other people talk about their surgeries or their chemotherapy, but you just want to be around people who understand.

Female, 29 years, thyroid cancer, ongoing therapies

Some have made lasting friendships within tightly knit online cancer communities, whereas others found that connecting with just a few people on the web helped combat isolation. Participants emphasized the importance of shared experiences; it is nice to vent to someone who understands. A young adult with breast cancer spoke about her *breasties* on the web:

Whatever is going on in your life, you feel like you can post there without judgment...Because I feel like a lot of times your friends and family don’t quite understand everything that your cancer sisters or your breasties would understand.

Female, 38 years, breast cancer, completed treatment

Sometimes, participants enjoyed just being together (virtually) with peers who understand their experiences, for example, doing a shared craft or activity through a video chat. Others emphasized the importance of connecting with peers who lived nearby and seeking out social media accounts of local chapters of national cancer organizations to do so. Social media also allows young adults with cancer to create new groups to fulfill unmet needs for seeking or sharing peer support. One participant created her own web-based organization and the corresponding Facebook group for Black women to connect and discuss unique experiences.

#### Mental Health and Emotional Benefits

Many participants discussed the emotional benefits of web-based social support. Seeing others’ self-disclosures about mental health or responses to their mental health challenges made their experiences feel more *normal*. Venting about fears and frustrations with peers was also helpful for decompressing and expressing themselves.

Seeing others’ positive cancer milestones or success stories (ie, “five years out from remission” or “I had a baby in spite of chemo”) helped some feel hopeful for their future. Gathering resources from online groups helped others feel in control of their cancer diagnosis. Humor can also be a helpful coping mechanism; young adults sought out funny, relatable posts from Instagram accounts such as @thecancerpatient, with the posts’ numerous likes and comments, to help them feel less alone.

Furthermore, social media provides young adults with cancer with individualized encouragement. Participants made friends on the web who would occasionally reach out to check in on their well-being and they would do the same in return. One participant described a tight-knit online group that regularly posted *mental health checks* for updates from each member. One participant explained the following:

Whenever I’m have anything that I am like mentally or emotionally struggling with, physically struggling with, any things that I feel are successes or roadblocks, like there are literally dozens of people now I feel like I can throw out a question to or an emotion to or something like that. And I’ve received so much just encouragement through those platforms that it’s really been a very healing factor for me...So I think it’s been a godsend for me.

Female, 32 years, sarcoma, completed treatment

#### Web-Specific Benefits

Although some participants preferred in-person support, others noted the specific benefits of web-based social support. On social media, participants do not face scheduling conflicts, can share without interrupting others, are not restricted to a number of questions, and can receive support throughout the day (and often night). Social media also gives more control—young adults can choose when to connect, view others’ profiles before connecting, choose how much information to disclose or engage with others’ emotions, and can easily leave unhelpful groups.

Social media also facilitates connection building in unique ways. Social media search capabilities helped participants find others with similar diagnoses. Young adults with cancer enjoyed getting a sense of others’ personalities through their social media profiles. Participants also referenced the ability to congregate large audiences on social media and the ease of use of already-familiar platforms for social support.

### Downsides of Web-Based Social Support

Young adults with cancer also face downsides and barriers for social support on social media, including disheartening content, participation burdens, others’ bad behavior, privacy concerns, and other shortcomings of social media platforms.

#### Disheartening Content

News about others’ declining health and the deaths of friends is one of the main downsides to web-based social support. Losing friends on the web can “hurt [their] hope” [Female, 29 years, breast cancer, ongoing therapies] or create a *hard and painful* experience that “adds to a little bit [of], you know, the heartache” [Female, 36 years, breast cancer]. Reading about others’ anxieties or negative outlooks—sometimes including worries that participants had not yet considered—was also discouraging. Similarly, *hard truth* posts (ie, photos of scars and harsh treatment realities), horror stories, and other unwanted information (ie, survival rates) heightened feelings of being overwhelmed. Reflecting on typical responses to new group members, one participant said the following:

Okay, like, back off a little bit, like, you know? Like they just started this process. This journey is very hard. You know, they don’t want to hear your horror story right away.

Female, 30 years, breast cancer, completed treatment

#### Participation Burden

Actively participating in web-based social support can also be emotionally taxing. Talking about cancer so often, being on the web at all times, and lending support can be draining, especially when feeling overwhelmed with one’s own treatment or recovery. Although posttreatment young adults often desire to support others in treatment, some feel that doing so is difficult. Providing this support can exacerbate the *survivor’s guilt*, especially if the person in treatment dies. Anticipating this experience made some posttreatment young adults hesitant to provide support on the web.

Other participants felt that supporting others in treatment forced them to relive their own traumatizing experiences. One participant explained the following:

I was a mentor for a couple people, and I actually had to pull away because I kept reliving what I went through every time they were going through their next step, and it just, kind of pulled on my heartstrings. It would just bother me when I heard someone had a worse-case scenario than I did.

Female, 38 years, breast cancer, ongoing therapies

Others encountered high volumes of requests for support. One participant described a web-based connection asking for near-constant support and sending countless messages even after stating that she was busy. Participants who regularly posted about their cancer on Twitter and Instagram received an overwhelming number of direct messages with cancer-related questions; answering quickly became burdensome.

#### Bad Behavior From Others

Beyond sharing terrifying stories with newly diagnosed young adults and unreasonable support demands, information sharing and giving advice can become troublesome. People often share false or questionable health information with a tone of authority, sometimes becoming pushy or insistent. Participants noted people on the web asking for (seeking) individual health recommendations that required consultation from a doctor. One participant provided the following anecdote:

I have a very rare tumor in my leg, and so one of the options if treatment didn’t work was amputation...And I just remember this one person also saying, like bombarding me..., “Why would you want to put yourself through being poisoned? Why would you want to put yourself through all of that? Just go get a consult, just go, amputation is the best option.” And that really weighed on me, especially while I was actively going through treatment. And these people’s opinions, they have, the ability to be like very aggressive or very harassing about them.

Female, 32 years, sarcoma, completed treatment

Another participant was bullied in a Facebook group for admonishing other members’ harsh behavior; they were criticizing a woman who disclosed her worst fear was losing her hair. A different participant complained about the unrealistically positive groups.

#### Privacy Concerns

Some young adults fear being treated differently, cancer-related stigma, and discrimination if employers knew about their cancer status. In light of these concerns, some participants preferred sharing private messages with other young adults with cancer rather than posting publicly. Others cited concerns about data security on platforms such as Facebook. In group contexts, participants worried someone might share information outside of the group or participate in a group video chat while others can hear. Finally, a few noted concerns about being *catfished* or connecting with someone who is pretending and people joining groups only to promote products or events.

#### Other Shortcomings of Social Media

Poor design features of social media were noted as shortcomings of web-based social support by over a third of participants. For example, long group threads that lacked organization (eg, posts not in time order) or notifications can be overwhelming, cause someone to miss potentially helpful posts, and hinder meaningful conversations:

I would miss things, and especially in the Facebook groups, they just, like, posts get jumbled up.

Female, 32 years, lung cancer, in treatment

Conversely, more often, participants complained about too many notifications:

Leading up to surgery, I was like psyching myself out. So, I kind of shut off the notifications on Facebook....

Female, 31 years, ovarian cancer, completed treatment

although, at the same time, they can be kind of overwhelming when you have a notification for every time anyone does anything, which kind of...disincentivizes you to kind of keep on [the app].

Male, 27 years, Hodgkin lymphoma, completed treatment

Other design flaws included apps and platforms that did not provide sufficient profile options or information. For example, apps that do not indicate whether someone is active or inactive on the site can prevent connections:

If I search for someone that’s like in their mid 30s, that had this diagnosis, but they haven’t logged on for like a month, then I’m not going to send them a friend request or I’m not going to send them a message of, like, “Hey, let’s like let’s be friends forever.” I would rather find someone else that has a totally different diagnosis, but that’s my friend, but that was on yesterday.

Female, 38 years, uterine endometrial cancer, completed treatment

Designs that exclude people with nonbinary gender identities by requiring users to select “male” or “female” were also disliked. One participant shared the following:

You feel very alone because like I would say a common LGBTQ feeling is alone. Cancer, alone. And when you put them together It’s even worse.

Male, 18 years, Hodgkin lymphoma, completed treatment

Similarly, another participant advocated for inclusive peer groups:

I think just being able to have that option [non-binary] is, like, the start of them at least feeling like they’re included in this. You do a medical intake and it’s male or female, like what, what if this person feels like they’re non-binary...there’s never a box for that.

Female, 38 years, breast cancer, completed treatment

In private online groups, such as Facebook groups, the number and variety of group members can present issues. Some participants liked big groups for crowdsourcing information; however, they found that they often did not make meaningful relationships in large groups:

I go on there from time to time, like, you know, we’ll talk to each other a little bit, ask questions, kind of share stories, things like that on Facebook, like in our own like private group that’s full like just thousands upon thousands of people all over the world.

Female, 24 years, Hodgkin lymphoma, completed treatment

In addition, diversity in big groups can make it difficult to find diagnosis-specific information:

I don’t ever post in there, and I don’t read a lot. And because there’s just so many people that it just seems too vague and overwhelming.

Female, 31 years, thyroid cancer, ongoing therapies

## Discussion

### Principal Findings

Many young adults with cancer turn to social media when they need peer-to-peer support. Experiences of social isolation among young adults with cancer have been well documented [3,4,6-9]. Social media holds promise to meet unmet support needs; most of our participants found that connecting with others on the web offered an important means of feeling less alone. They went on the web seeking shared connections, using both general platforms such as Facebook and Instagram as well as specialized spaces such as forums hosted by Caring Bridge or Stupid Cancer to help meet their needs.

These digital resources help young adults gather information on key topics tailored to their diagnoses, life stages, and communication preferences. Among our participants, it was clear that social media can help access essential information while working around the inherent problems of navigating a cancer diagnosis. The range of platforms, technology types, and online support communities can offer multiple avenues of support, helping to efficiently meet different needs at different times. For example, immunocompromised individuals do not have the option of attending an in-person support group, so a private Facebook group or Twitter chat can offer much-needed information or help them cope. Doing so likely meets support needs that cannot be met by others in their support network; cancer-specific social media is used more among survivors with fewer close support resources [42].

The larger size of many social media groups—on Facebook and elsewhere—makes it increasingly possible for individuals to find someone with a similar diagnosis or a specific personal challenge [43,44]. Social media becomes more useful as the diversity and number of users increase (ie, network effects); more peers lead to more opportunities to connect to similar experiences [45]. The scale of general audience social media communities allows for the increased possibility of a user finding, for example, someone else with the same rare genetic mutation, food allergy, or relationship status that one would be unlikely to come across in the face-to-face world through hospital-organized or geography-based programs. Participants in this project perceived value in being able to crowdsource instrumental support, particularly without concern for specificity of issue, geography, or time; however, large group discussions can be overwhelming, making it difficult to build close relationships with others.

Convenience is critical—young adults with cancer desire support that meets them where they are and when they need it [13,14]. Social media groups also allow these young adults to have additional agency over the depth, timing, and frequency of cancer-related social interactions. Participants in most social media groups can maintain a degree of anonymity, if desired, and choose how much they share, as well as how often they participate, all in ways substantially different from in-person support. In addition, as several participants mentioned, there is a special and appreciated opportunity to control the pace of relationships such that established and safe web-based connections have the chance to move to other communication channels and degrees of depth when both parties agree.

Along with the opportunity to search for difficult-to-find information and receive emotional support, participants also noted that the opportunity to help others is a significant benefit of these online groups. Giving back offers a chance to build esteem and shift focus from one’s own challenges while getting the emotional and mental health benefits of participating in mutual sharing [46,47].

Social support via social media comes with challenges that also offer lessons about how to best create, manage, and suggest these resources for young adults with cancer. Participants mentioned downsides such as the difficulty of seeing others struggle or the difficulty of being connected to the cancer community nonstop, as well as problematic group behavior of asking for specific advice without being willing to engage a health care professional. Young adults with cancer are challenged with dealing with exaggerated, unsupported, and misleading information on the web and across social media [48,49]. Many of these challenges can also exist with in-person relationships as well; however, the access and opportunity to engage in interactions on the web is greater, so the considerations are slightly different, where one’s digital presence does not fade, and reminders of pain and loss are harder to avoid [50].

Considering these potential downsides, online groups have the possibility of using design features and moderation to change norms, reduce these burdens, and maximize support for users. Improved outcomes could come from design elements, such as tools, to flag problematic posts. In-platform prompts (eg, “want to review?”) can encourage communities to intervene to reduce harmful content [51] and provide individuals with effective prompts to rethink or edit what they share [52,53]. Platforms should also leverage features for peer matching based on emotional needs (eg, “I would like to offer support” or “I am just here to observe”*)* so people can control their emotional labor; the ability to *signal* availability, without the cognitive or emotional burden of an explanation, can function as a socially acceptable warning or active invitation for support requests. Similarly, clearly stated community standards to reduce offensive or misleading content; tools to allow for content flagging, reporting, and shadow banning (ie, hiding posts or comments from future views) to allow the communication to participate in reducing harmful content; and active platform moderators to intervene could aid in ensuring a consistent, useful culture. Finally, newer technologies such as chatbots could reduce the downsides of social media-based support by taking on emotional engagement and coaching individuals in how to most constructively discuss their circumstances.

### Limitations and Future Directions

This study had some limitations. Young adults responded to recruitment emails or social media posts shared directly (or indirectly through likes) from the Stupid Cancer community; thus, participants may differ from other young adults with cancer who are less digitally connected to strong cancer advocacy organizations. Future extensions of this work should use different recruitment strategies (eg, snowball sampling, recruitment from other organizations, and paid social media recruitment) to hear from less connected populations for other, and potentially greater, peer support needs. How young adults may be receiving support from sources other than social media and thus feeling less inclined to connect is also important to consider in further research. The sample was also primarily female, identified as White, and breast cancer was the most common diagnosis (15/45, 33%). More representative and inclusive samples of young adults of varying ages, balanced for gender, and with a variety of cancer diagnoses are needed to understand unique needs and challenges. It would also be fruitful to contribute to emerging evidence [54,55] and investigate whether family and friends use social media in similar ways to support young adults with cancer in their lives or for their own caregiver support. Similarly, hearing from health care providers and clinical teams about their experiences recommending resources or using social media to support young adult patients is needed to better understand how and when peer support fits within clinical care [49]. In addition, our interviews were conducted before the scale and the impact of COVID-19 were known. Although some participants referenced the impact of the pandemic on their web-based support use organically, participants largely focused on life before the pandemic. Follow-up studies are needed to determine novel or evolving benefits and downside of web-based support during and after the pandemic, where many social connections have a greater emphasis on technology-based interactions.

With growing evidence for the benefits of web-based cancer support, in spite of downsides, there is a need to determine how to best encourage young adults with cancer to try out social media support options that may work for them. To do that, we can focus more on *when* young adults use social support [37], specifically when they initiate or engage with web-based social support and how their use changes through their cancer timeline, as well as how clinicians can better highlight perceived benefits directly to patients and advocate for the management of downsides.

### Conclusions

Social media provides beneficial social support for young adults to connect with peers about cancer experiences. More social media options where young adults can comfortably share their concerns are needed. Barriers to social media support could be reduced through content moderation, customizable features for content flagging and discussion size, flexible profile creation to signal identity and support desires, and secure platforms with large user bases to facilitate meaningful connections for shared, often diagnosis-specific experiences.
